# Advancing Fingertip Regeneration: Outcomes from a New Conservative Treatment Protocol

**DOI:** 10.3390/jcm13133646

**Published:** 2024-06-21

**Authors:** Daihun Kang

**Affiliations:** Department of Plastic and Reconstructive Surgery, Ewha Womans University Seoul Hospital, Seoul 03760, Republic of Korea; gpk1234567@naver.com; Tel.: +82-10-4724-1419

**Keywords:** artificial dermis, fingertip, fingerprint, Pelnac^®^, reconstruction, regeneration, semi-occlusive, volar

## Abstract

**Background** Fingertip injuries with volar pulp tissue defects present a significant challenge in management. This study aimed to evaluate the efficacy of a conservative treatment protocol using artificial dermis and semi-occlusive dressings for these injuries. **Methods** A single-center, prospective study was conducted on 31 patients with fingertip injuries involving volar pulp defects. The treatment protocol included wound debridement, application of artificial dermis (Pelnac^®^), and a semi-occlusive dressing (IV3000^®^). The outcomes were assessed using subjective questionnaires and objective measures, including fingerprint regeneration, sensory function, pain, and cosmetic appearance. **Results** The mean treatment duration was 45.29 days (SD = 17.53). Complications were minimal, with only one case (3.22%) directly attributable to the treatment. Fingerprint regeneration was considerable (mean score = 2.58, SD = 0.67). The sensory disturbances were minimal, with no significant differences across injury types. Post-treatment pain was low (mean = 0.45, SD = 0.67), and cosmetic satisfaction was high (mean = 4.09, SD = 0.94). The overall patient satisfaction was high (mean = 4.41, SD = 0.67), regardless of injury severity. **Conclusions** The conservative treatment protocol using artificial dermis and semi-occlusive dressings is a promising strategy for managing fingertip injuries with volar pulp defects. This approach minimizes surgical morbidity and achieves excellent functional and aesthetic outcomes.

## 1. Introduction

The human hand, a masterpiece of intricate anatomy and functional harmony, is our primary tool for exploring and interacting with the world around us. At the very tips of our fingers lies the volar pulp—a highly specialized structure essential for fine-touch sensation, grip stability, and precise manipulation [1,2]. This complex interplay of sensory and motor functions is made possible by the rich neurovascular network and specialized sensory receptors within the pulp [2]. However, the same complexity that endows the fingertips with their remarkable abilities also renders them particularly vulnerable to injury. Fingertip injuries involving volar pulp tissue defects can lead to significant functional impairment, sensory disturbances, and cosmetic disfigurement, ultimately impacting the patient’s quality of life [3,4].

Traditionally, surgical approaches, such as local flaps, skin grafts, and free tissue transfers, have been employed to manage fingertip injuries with volar pulp defects [3,5,6,7,8,9]. While these techniques aim to restore the soft tissue envelope and preserve finger length, they are often associated with donor site morbidity, prolonged immobilization, and the need for multiple surgeries [3,4,10]. Moreover, these surgical interventions may not adequately restore the intricate sensory and biomechanical properties of the native pulp tissue, leading to suboptimal functional outcomes [4,11,12,13].

In pursuit of more effective and less invasive treatment options, there has been a growing interest in conservative approaches that harness the body’s inherent regenerative potential [14]. The use of artificial dermal substitutes, such as Pelnac^®^ (Gunze Limited, Kyoto, Japan), has shown promising results in managing various soft tissue defects [15,16,17,18]. These biomaterials provide a three-dimensional scaffold that mimics the natural extracellular matrix, facilitating cell migration, proliferation, and differentiation [19,20].

While previous studies have investigated the use of artificial dermis or semi-occlusive dressings separately in the treatment of fingertip injuries [18,21,22,23,24], the potential synergistic effects of combining these two approaches have not been extensively explored. Drawing inspiration from the adage that the whole is greater than the sum of its parts, this study aims to evaluate the efficacy of a novel conservative treatment protocol that combines the use of artificial dermis (Pelnac^®^) with a semi-occlusive dressing (IV3000^®^) in managing fingertip injuries with volar pulp tissue defects.

The rationale behind this combination is that the artificial dermis provides a structured scaffold for tissue regeneration, while the semi-occlusive dressing maintains a moist wound environment and allows for gas exchange [19,22,24,25]. The author hypothesizes that this synergistic approach will promote the regeneration of vascularized and innervated pulp tissue, leading to superior functional and aesthetic outcomes compared to traditional surgical interventions or conservative treatments using either artificial dermis or semi-occlusive dressings alone.

The potential benefits of this novel treatment protocol are multifaceted. First, it may reduce healing time and minimize complications associated with purely conservative treatments [20]. Second, it could lead to improved long-term functional and aesthetic outcomes by supporting the regeneration of complex tissue architecture [20,25,26]. Finally, despite the higher initial cost, this approach may prove to be cost-effective in the long run by achieving superior clinical outcomes and reducing the need for additional interventions [18].

To test this hypothesis and evaluate the effectiveness of this new combination protocol, a prospective study was conducted with the following primary objectives:

1. Assess the regeneration of volar pulp tissue, including the restoration of fingerprint pattern, sensory function, and cosmetic appearance.
2. Evaluate the impact of injury characteristics, such as bone or tendon exposure, on treatment outcomes and duration.
3. Investigate the incidence of complications and patient-reported outcomes, including pain, sensory disturbances, and overall satisfaction.
4. Compare the treatment duration and outcomes with those reported in previous studies utilizing artificial dermis or semi-occlusive dressings separately for fingertip reconstruction.

This study aims to provide valuable insights into the efficacy and feasibility of this novel treatment for managing fingertip injuries with volar pulp tissue defects.

## 2. Materials and Methods

**Figure 1:**
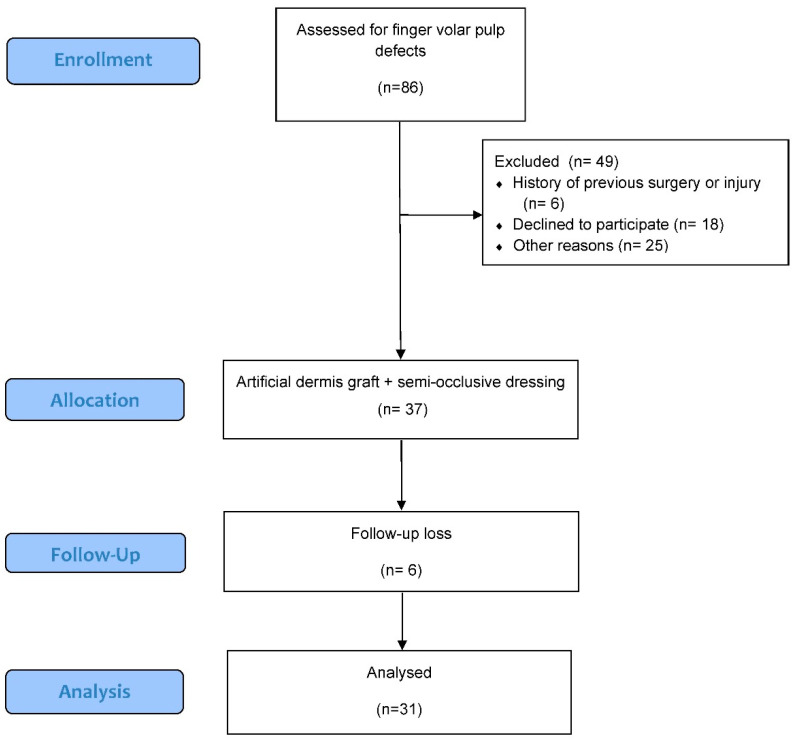
The flow diagram of this study.

A single-center, prospective study was conducted at our institution, which was approved by the Institutional Review Board (IRB) of the Catholic Kwandong University International St. Mary’s Hospital (Approval No. 19 year IRB069, Registration No. IS19EISE0072). This study was performed in strict adherence to ethical guidelines. Written informed consent was obtained from all participants or their guardians prior to participation in this study. The study period was from December 2019 to December 2022, focusing on patients presenting with volar pulp tissue defects of the fingertip (Figure 1).

To ensure robust statistical power, a power analysis was conducted prior to patient recruitment. The analysis determined that a minimum of 30 patients in total was necessary to adequately power this study, assuming an alpha of 0.05, a power of 0.80, and an effect size of 0.5. This sample size was chosen to ensure that the study results would be statistically significant and replicable in similar clinical settings. Written informed consent was obtained from all participants involved in this study.

### 2.1. Inclusion and Exclusion Criteria

The inclusion criteria included patients with volar pulp tissue defects who provided informed consent and were unsuitable for immediate replantation. The exclusion criteria were immediate surgical intervention needs, history of surgery or injury to the affected finger, or underlying conditions impairing wound healing.

### 2.2. Treatment Protocol

**Figure 2:**
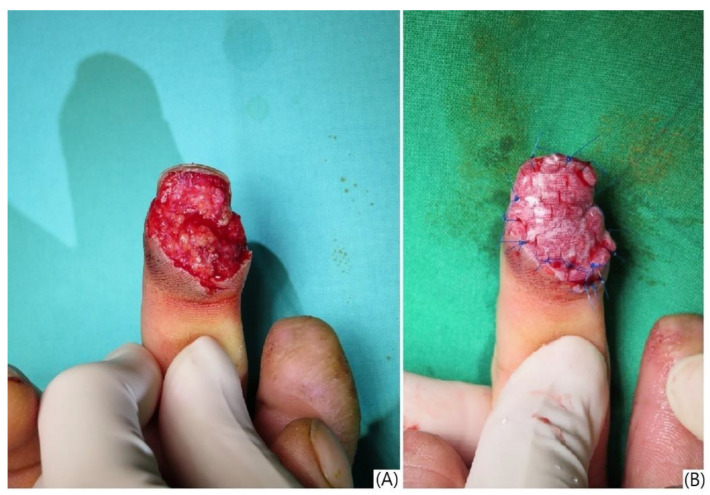
Application of artificial dermis and semi-occlusive dressing for fingertip injury. (**A**) Preoperative view of a middle finger pulp injury; (**B**) intraoperative view showing the application of artificial dermis (Pelnac^®^) to the defect, followed by a semi-occlusive dressing (IV3000^®^).

All patients underwent wound debridement, irrigation, and hemostasis under digital nerve block in the operating room. The artificial dermis (Pelnac^®^; Gunze Limited, Kyoto, Japan), a bilayer membrane composed of an outer silicone layer and an inner collagen sponge layer with a thickness of 3 mm, was soaked in saline, trimmed to cover the defect, and secured with 4-0 nylon sutures (Figure 2). A semi-occlusive dressing (IV3000^®^; Smith & Nephew, Watford, UK), a transparent, waterproof, and breathable polyurethane film dressing, was then applied over the artificial dermis. For patients with excessive oozing, the dressing was changed the following day at the outpatient clinic. In other cases, the first dressing change was performed five days post-operation.

During dressing changes, the finger was soaked in saline for 15 min to minimize damage to the Pelnac^®^ during IV3000^®^ removal. The wound and surrounding area were gently irrigated with saline to prevent infection, and a new IV3000^®^ dressing was applied. The Pelnac^®^ consists of two layers: an inner atelocollagen sponge and an outer silicone layer. After approximately two weeks, the silicone layer was removed, exposing the raw surface. From this point onwards, only the IV3000^®^ dressing was used to cover the wound, creating a moist environment conducive to epithelialization. This process was repeated at five-day intervals until complete epithelialization was achieved, which was defined as the complete coverage of the wound with epithelial tissue without any remaining raw surface. The duration from the initial application of the artificial dermis to complete wound healing was recorded.

### 2.3. Outcome Measures

After complete wound healing, patients were asked to complete a subjective questionnaire assessing the following outcomes:

- Cosmetic satisfaction: rated on a scale from 1 to 5, with higher scores indicating greater satisfaction with the appearance of the treated finger.
- Sensory impairment: rated on a scale from 0 to 5, with lower scores indicating less sensory loss.
- Sensory hypersensitivity: rated on a scale from 0 to 5, with lower scores indicating less discomfort or abnormal sensations.
- Pain: assessed using a visual analog scale (VAS) ranging from 0 to 5, with lower scores indicating less or no pain.
- Overall satisfaction: rated on a scale from 1 to 5, with higher scores indicating greater satisfaction with all aspects of the treatment and recovery process.

To objectively evaluate the pulp tissue reconstruction, the degree of fingerprint regeneration was assessed on a scale from 0 to 3, with higher scores indicating better regeneration. This scoring system was developed specifically for this study to quantify the extent of fingerprint recovery: 0 indicating no regeneration, 1 indicating partial regeneration, 2 indicating significant regeneration with some irregularities, and 3 indicating nearly complete regeneration with clear fingerprint patterns. 

The outcome assessments were performed by two independent, trained assessors who were blinded to the patients’ treatment allocation. The assessors underwent a calibration exercise to ensure consistency and reliability in their evaluations.

Complications, including hook nail deformity and onychomycosis, were recorded throughout the study period. The outcome assessments and follow-up visits were conducted at 1, 3, and 6 months post-treatment.

### 2.4. Statistical Analysis

The Shapiro–Wilk test was employed to verify the normality of the data distribution. Treatment duration, which followed a normal distribution, was described using means and standard deviations and analyzed using independent sample *t*-tests to compare:

- Patients with and without bone exposure.
- Patients with and without tendon exposure.
- Patients with both bone and tendon exposure versus those with neither.

Non-normally distributed variables, including fingerprint regeneration scores, cosmetic satisfaction, sensory impairment, sensory hypersensitivity, pain, and overall satisfaction, were described using medians and interquartile ranges. These variables were compared using the Mann–Whitney *U* test, a non-parametric test, across different patient groups.

Statistical significance was established at a *p*-value of less than 0.05. All statistical analyses were performed using IBM SPSS Statistics 29.0.2.0 (IBM Corp., Armonk, NY, USA).

## 3. Results

### 3.1. Participant Demographics and Injury Characteristics

A total of 31 patients with fingertip injuries were included in this study, conducted between January 2017 and December 2021. The study cohort consisted of 25 males (80.6%) and 6 females (19.4%), with a mean age of 43.29 years (range, 18–72 years). The distribution of the affected fingers is presented in Table 1. 

Key finding: the study population was predominantly male, and the index finger was the most commonly injured one.

### 3.2. Treatment Protocol and Healing Duration

**Figure 3:**
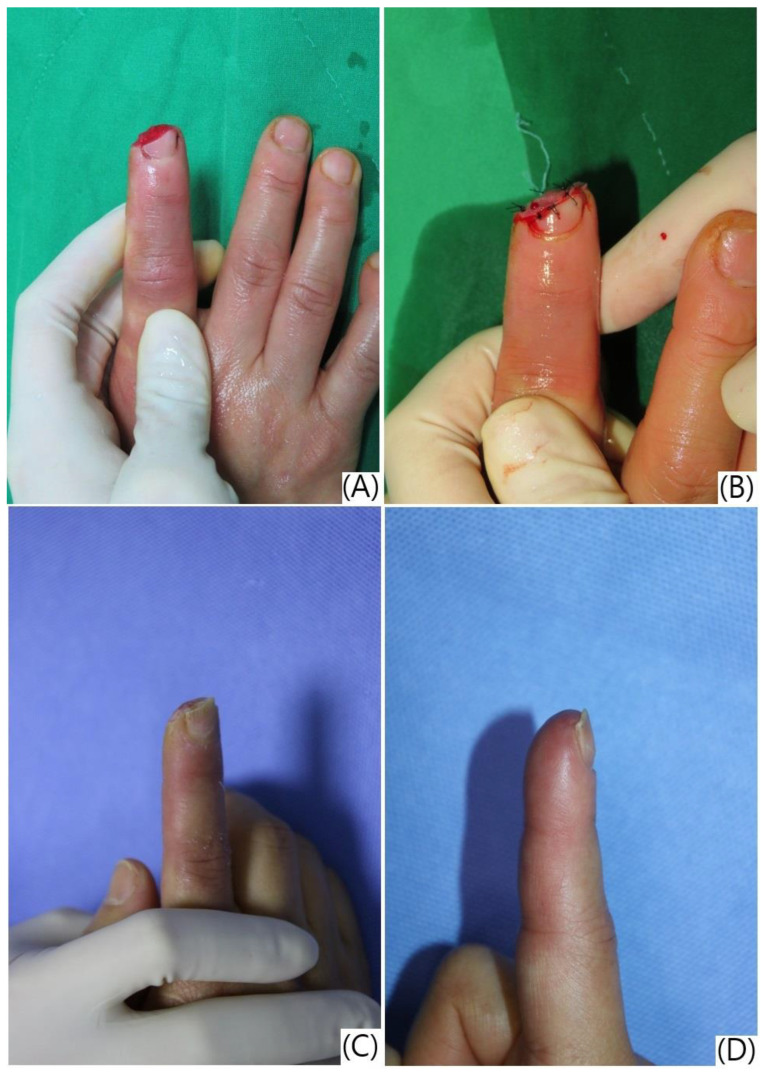
Treatment progression and outcomes following artificial dermis application and semi-occlusive dressing for a fingertip injury. (**A**) Pre-operative view of the right index fingertip injury with distal phalanx and soft tissue defect; (**B**) immediate post-operative view after debridement and application of artificial dermis; (**C**) five weeks post-treatment, showing the regeneration of pulp tissue and early restoration of the fingerprint pattern under the semi-occlusive dressing; (**D**) seven weeks post-treatment, demonstrating complete wound healing with well-formed fingerprint ridges, restored sensation, minimal pain, and satisfactory cosmetic appearance.

Among the 31 patients, 9 (29.03%) had soft tissue injuries without bone exposure, while 22 (70.97%) had injuries with bone exposure (Figure 3). The mean treatment duration for all patients was 45.29 days (SD = 17.53).

Key finding: the mean treatment duration was 45.29 days (SD = 17.53), with no significant differences based on bone or tendon exposure, although cases with tendon exposure required a slightly longer treatment duration compared to those with bone exposure.

### 3.3. Complications and Clinical Outcomes

**Figure 4:**
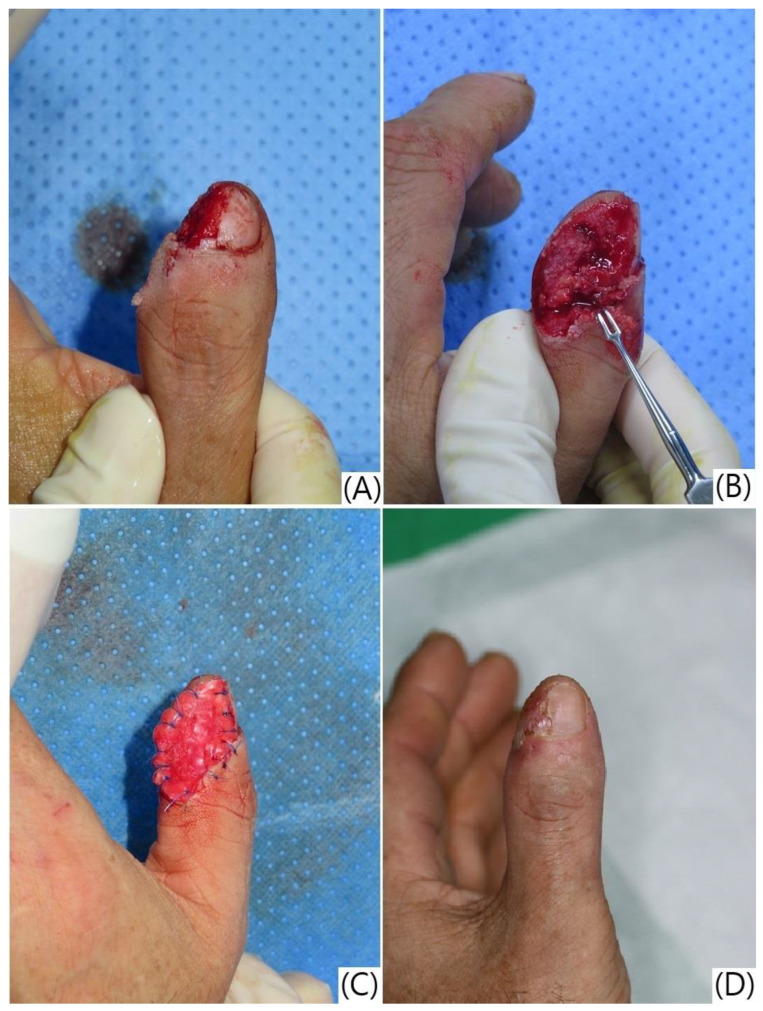
Reconstruction of a thumb tip defect using artificial dermis application and semi-occlusive dressing, resulting in nail splitting. (**A**,**B**) Pre-operative pictures showing a soft tissue defect of the right thumb side wall with tendon and bone exposure; (**C**) appearance after artificial dermis grafting; (**D**) follow-up photograph 7 weeks after the treatment, demonstrating good healing of the defect site but with observable nail splitting.

**Figure 5:**
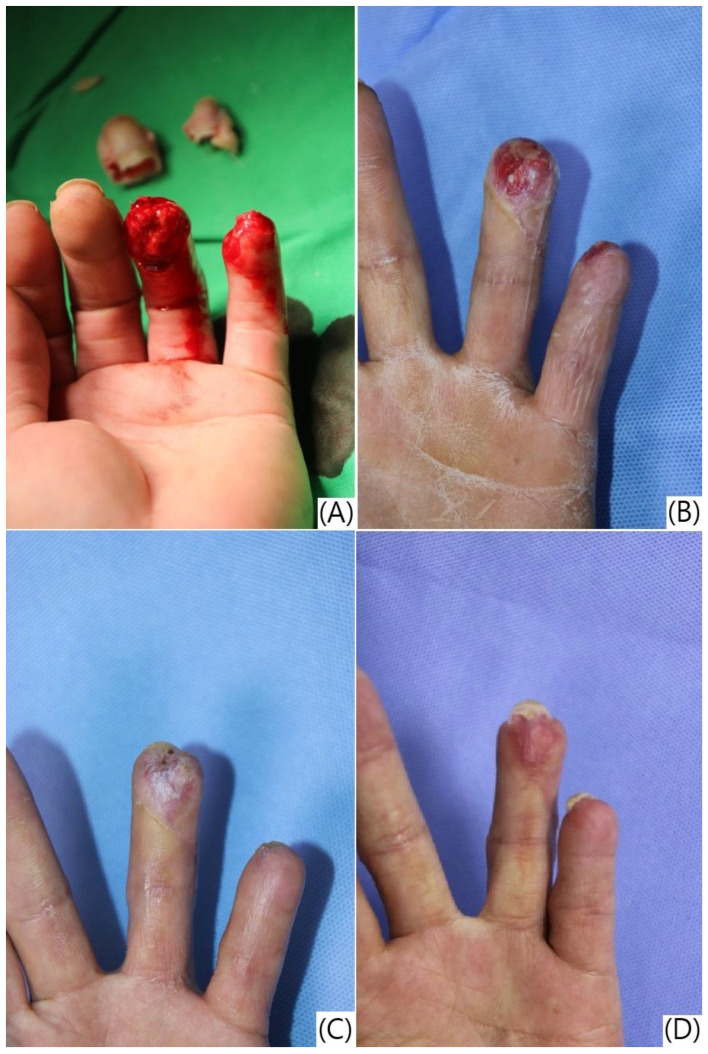
Clinical progression of fingertip regeneration following treatment with artificial dermis and semi-occlusive dressing, demonstrating variability in outcomes. (**A**) Pre-operative view of severe injuries to the left ring and little fingers with complete soft tissue loss and exposed distal phalanges; (**B**) four weeks post-treatment, showing significant granulation tissue formation and coverage of the exposed bone; (**C**) seven weeks post-treatment, demonstrating slower progression of wound healing, with no exposed bone and partial restoration of the fingerprint ridges. However, trophic changes in the nail bed are evident due to the shortened distal phalanges; (**D**) ten weeks post-treatment, revealing complete wound healing with residual scarring on the ring finger and near-complete regeneration of the fingerprint on the little finger. Hook nail deformities are present in both fingers as a consequence of the shortened distal phalanges, which could not be lengthened by the treatment protocol.

Complications were reported in nine patients (29.03%), including hook nail deformity (n = 6, 19.35%), scar contracture (n = 1, 3.22%), onychomycosis (n = 1, 3.22%), and nail splitting (n = 1, 3.22%). Only one complication (3.22%) of nail splitting was directly related to the treatment (Table 2, Figure 4 and Figure 5).

Key finding: the treatment demonstrated a low complication rate, with only one case (3.22%) of nail splitting directly attributable to the procedures used in this study.

### 3.4. Fingerprint Regeneration

The mean fingerprint regeneration score was 2.58 (SD = 0.67) on a scale from 0 to 3, suggesting significant restoration of the fingerprint pattern in most patients.

Key finding: fingerprint regeneration was considerable (mean score = 2.58, SD = 0.67) across all patients, with no statistically significant differences based on bone or tendon exposure (see Table 3 for detailed statistics).

### 3.5. Sensory Assessment

#### 3.5.1. Hypoesthesia

The mean hypoesthesia score for all 31 patients was 0.09 (SD = 0.39), indicating minimal sensory deficits.

Key finding: hypoesthesia was minimal (mean = 0.09, SD = 0.39), with no significant differences across various injury types.

#### 3.5.2. Hyperesthesia

The mean hyperesthesia score for all 31 patients was 0.25 (SD = 0.57), indicating minimal sensory disturbances.

Key finding: hyperesthesia was minimal (mean = 0.25, SD = 0.57), with no significant differences across various injury types.

### 3.6. Pain Assessment

The mean pain score for all 31 patients was 0.45 (SD = 0.67), indicating minimal post-treatment pain.

Key finding: post-treatment pain was minimal (mean = 0.45, SD = 0.67), with no significant differences across various injury types.

### 3.7. Cosmetic Assessment

The mean cosmetic satisfaction score for all 31 patients was 4.09 (SD = 0.94), indicating high satisfaction with the cosmetic outcomes.

Key finding: cosmetic satisfaction was high (mean = 4.09, SD = 0.94), with no significant differences across various injury types.

### 3.8. Overall Satisfaction

The mean overall satisfaction score for all 31 patients was 4.41 (SD = 0.67), indicating high satisfaction with the treatment outcomes.

Key finding: overall treatment satisfaction was high (mean = 4.41, SD = 0.67), with no significant differences across various injury types (Table 3).

## 4. Discussion

This prospective study demonstrates the efficacy of a conservative treatment protocol combining artificial dermis (Pelnac^®^) and semi-occlusive dressings (IV3000^®^) in managing fingertip injuries with volar pulp tissue defects. The findings underscore the potential of this synergistic approach for achieving favorable clinical outcomes while minimizing surgical morbidity and hospitalization, aligning with the growing emphasis on patient-centered care in reconstructive surgery [27,28].

The study cohort exhibited substantial fingerprint regeneration, with slightly better scores in patients with bone-exposed injuries. This improvement could be attributed to the regenerative capabilities of the periosteum and bone marrow cells, as well as the inflammatory response triggering the recruitment of mesenchymal stem cells and growth factors [29,30]. These findings suggest that the conservative treatment protocol, which harnesses the body’s inherent regenerative mechanisms, may be particularly beneficial for patients with bone-exposed fingertip injuries.

The treatment duration in the current study (45.29 days, SD = 17.53) was slightly longer than those reported by Namgoong et al. [18] and Wang et al. [12], who also used artificial dermis for fingertip defects with bone exposure. This prolonged duration may be attributed to the unique combination of artificial dermis and semi-occlusive dressing, which likely influences the complex regenerative processes within the wound bed. Despite the longer treatment duration, the current study achieved high patient satisfaction, good sensory recovery, and favorable cosmetic outcomes, consistent with the key outcomes reported in other studies (Table 4). However, the heterogeneity in study designs, sample sizes, and specific interventions among these studies makes direct comparisons challenging.

The consistently low pain levels across all subgroups suggest effective pain management by the treatment protocol, possibly due to the anti-inflammatory properties of the artificial dermis and the protective barrier provided by the semi-occlusive dressing [31]. However, the conclusion of a “low pain level” should be interpreted cautiously, as the sensory evaluation was conducted immediately after epithelialization and may not fully capture long-term pain levels and nerve recovery [32].

This study has several limitations that warrant careful consideration. First, the absence of a surgical control group may affect the generalizability of the findings. This decision was guided by multiple factors: the inherent drawbacks of surgical interventions [6,7,8,10,13,33], encouraging outcomes from conservative approaches in prior studies [12,14,16,17,21,22,23,24,34,35,36], and significant recruitment challenges due to patients’ strong preference for less invasive treatments [37]. Moreover, based on the author’s experience, the toe pulp free flap was identified as the most comparable surgical technique to the proposed conservative treatment. However, its inclusion in this study was impractical given the highly specialized nature of the procedure and the significantly lower number of eligible patients, making it unfeasible to create a control group for this technique. 

**Table 4:** Comparison of treatment duration with previous studies utilizing artificial dermis for fingertip reconstruction.

Study	Sample Size	Injury Type	Method	Treatment Duration (days)	Key Outcomes
Current Study	31	Volar pulp defects with bone and/or tendon exposure	Pelnac^®^	45.29 ± 17.53	High patient satisfaction, good sensory recovery, and cosmetic outcomes
Wang et al., 2022 [12]	24	Fingertip defects with bone exposure	Pelnac^®^	28~42	Improves appearance and function, and decreases the need for stump trimming in amputated fingers.
Namgoong et al., 2020 [18]	23	Fingertip defects with bone exposure	Tissue-engineered artificial dermis	34.0 ± 4.9	Superior functional and aesthetic outcome compared to artificial dermis graft
Hoigné et al., 2014 [23]	19	Fingertip amputations(Ishikawa zones II–III)	OpSite^®^ Flexifix^®^ (Richardson Healthcare Ltd., Borehamwood, UK) dressing	21~56	Good sensory recovery and cosmetic outcomes
Boudard et al., 2019 [38]	19	Fingertip amputations (M and D * zones I–III)	Semi-occlusive dressing (Tegaderm^®^, 3M Company, St. Paul, MN, USA)	30.1 ± 7	Good functional and cosmetic outcomes
Mennen and Wiese, 1993 [36]	200	Various fingertip injuries	Semi-occlusive dressing (OpSite^®)^	20–30	Good functional and cosmetic outcomes, low complication rate

* M & D: Merle and Dautel.

Another important limitation is the lack of objective sensory recovery assessments, such as the two-point discrimination test, which could have provided a more comprehensive evaluation of sensory outcomes [39]. Additionally, the relatively short follow-up period restricts the ability to fully assess the long-term durability and efficacy of the treatment. Addressing these limitations in future research will be crucial for validating and extending the findings of this study. 

To address these limitations and further advance the understanding of conservative approaches for managing fingertip injuries, future research should focus on the following areas:

1. Conducting well-designed, randomized controlled trials comparing conservative treatments with surgical interventions, using standardized outcome measures and longer follow-up periods to assess long-term efficacy, durability, pain levels, and nerve recovery.
2. Performing comparative studies on different dermal substitutes and dressing materials to identify the most effective combinations that minimize treatment time while maximizing regenerative outcomes.
3. Incorporating objective sensory assessments, such as the two-point discrimination test, alongside subjective evaluations to provide a more comprehensive understanding of sensory recovery

## 5. Conclusions

This study demonstrates the potential of a novel conservative treatment protocol combining artificial dermis and semi-occlusive dressings in managing fingertip injuries with volar pulp tissue defects. Despite its limitations, such as the absence of a surgical control group and the relatively short follow-up period, the approach achieves favorable clinical outcomes while minimizing surgical morbidity and aligning with patient-centered care principles. The results suggest that this synergistic combination may offer a promising alternative to traditional surgical interventions for managing fingertip injuries, particularly in patients with bone-exposed defects. However, further research with longer follow-up periods, objective sensory assessments, and comparative studies is needed to establish the long-term efficacy, durability, and superiority of this approach over existing surgical techniques.

## Figures and Tables

**Table 1:** Participant demographics and injury characteristics.

Characteristic	Total (N = 31)	Bone Exposure	No Bone Exposure	Tendon Exposure	No Tendon Exposure
Gender					
Male	25 (80.6%)	17	8	8	17
Female	6 (19.4%)	5	1	2	4
Age (years)					
Mean ± SD	43.29 ± 15.00	43.11 ± 17.22	43.36 ± 14.44	40.50 ± 14.73	44.61 ± 15.30
Affected Fingers					
Thumb	3 (9.7%)	2	1	3	0
Index	13 (41.9%)	8	5	8	4
Middle	6 (19.4%)	4	2	4	2
Ring	8 (25.8%)	6	2	4	4
Little	5 (16.1%)	3	2	3	2

**Table 2:** Complications and their management.

Complication	No. of Patients (%)	Management
Hook nail deformity	6 (19.35%)	Observation and patient education
Scar contracture	1 (3.22%)	Steroid injection and silicone gel ointment
Onychomycosis	1 (3.22%)	Antifungal medication
Nail splitting	1 (3.22%)	Observation and patient education

**Table 3:** Subgroup analysis of outcome measures based on injury characteristics.

Outcome Measure	Bone Exposure	Tendon Exposure	Combined Exposure
Treatment duration (days)	44.11 ± 10.48 (without) vs. 45.77 ± 19.91 (with) (*p* = 0.131)	41.71 ± 15.67 (without) vs. 52.80 ± 19.65 (with) (*p* = 0.512)	43.63 ± 11.09 (neither) vs. 53.33 ± 20.76 (both) (*p* = 0.143)
Fingerprint regeneration score †	2.44 ± 0.88 (without) vs. 2.63 ± 0.58 (with) (*p* = 0.781)	2.57 ± 0.67 (without) vs. 2.60 ± 0.69 (with) (*p* = 0.917)	2.37 ± 0.91 (neither) vs. 2.55 ± 0.72 (both) (*p* = 0.815)
Hypoesthesia score §	0.22 ± 0.66 (without) vs. 0.04 ± 0.21 (with) (*p* = 0.426)	0.09 ± 0.43 (without) vs. 0.10 ± 0.31 (with) (*p* = 0.968)	0.25 ± 0.70 (neither) vs. 0.11 ± 0.33 (both) (*p* = 0.963)
Hyperesthesia score §	0.11 ± 0.33 (without) vs. 0.31 ± 0.64 (with) (*p* = 0.382)	0.28 ± 0.64 (without) vs. 0.20 ± 0.42 (with) (*p* = 0.702)	0.12 ± 0.35 (neither) vs. 0.22 ± 0.44 (both) (*p* = 0.743)
Pain score (VAS) §	0.44 ± 0.52 (without) vs. 0.45 ± 0.73 (with) (*p* = 0.781)	0.38 ± 0.58 (without) vs. 0.60 ± 0.84 (with) (*p* = 0.633)	0.37 ± 0.51 (neither) vs. 0.55 ± 0.88 (both) (*p* = 0.743)
Cosmetic satisfaction score ¶	4.22 ± 0.97 (without) vs. 4.04 ± 0.95 (with) (*p* = 0.654)	4.14 ± 0.91 (without) vs. 4.00 ± 1.05 (with) (*p* = 0.787)	4.12 ± 0.99 (neither) vs. 3.88 ± 1.05 (both) (*p* = 0.673)
Overall satisfaction score ¶	4.66 ± 0.70 (without) vs. 4.31 ± 0.64 (with) (*p* = 0.174)	4.47 ± 0.67 (without) vs. 4.30 ± 0.67 (with) (*p* = 0.492)	4.62 ± 0.74 (neither) vs. 4.22 ± 0.66 (both) (*p* = 0.236)

Data presented as without exposure vs. with exposure, *p* > 0.05 for all comparisons (independent samples *t*-test). † Assessed on a scale from 0 to 3, with higher scores indicating better regeneration. Data presented as without exposure vs. with exposure, *p* > 0.05 for all comparisons (Mann–Whitney U test). § Rated on a scale from 0 to 5, with lower scores indicating less sensory loss, discomfort or pain. ¶ Rated on a scale from 1 to 5, with higher scores indicating greater satisfaction.

## Data Availability

The data presented in this study are not publicly available due to privacy and ethical restrictions, as the research involves sensitive patient information.
